# Efficacy of YAG Laser Embolysis in Retinal Artery Occlusion

**DOI:** 10.12669/pjms.37.1.3196

**Published:** 2021

**Authors:** Mohammad Asim Mehboob, Asfandyar Khan, Ahsan Mukhtar

**Affiliations:** 1 Dr. Mohammad Asim Mehboob, FCPS (Ophth), FICO, FRCS (Glasgow), MRCSEd (Ophth) CMH Gujranwala, Gujranwala, Pakistan; 2 Dr. Asfandyar Khan, FCPS (Ophth). Registrar Vitreo-Retinal Ophthalmology, Armed Forces Institute of Ophthalmology, Rawalpindi, Pakistan; 3 Dr. Ahsan Mukhtar, FCPS (Ophth), FCPS (Vitreo-retinal Ophth) FRCS (Glasgow) Armed Forces Institute of Ophthalmology, Rawalpindi, Pakistan

**Keywords:** Retinal artery occlusion, Nd:YAG laser embolysis, Vitreous hemorrhage

## Abstract

**Objective:**

To compare the anatomical and functional success between conventional medical method and Neodymium-Doped Yttrium Aluminum Garnet (Nd:YAG) laser embolysis in retinal artery occlusion.

**Methods:**

This randomized control trial was conducted at Armed Forces Institute of Ophthalmology (AFIO) Rawalpindi from July 2018 to May 2020. A total of 14 eyes of 14 patients were received with fovea involving branch or hemiretinal artery occlusion within 24 hours of onset of symptoms. They were divided randomly in two groups. Initial treatment was given to all cases, and seven eyes received Nd:YAG laser treatment for embolysis. Both groups were analysed for anatomical success (reperfusion) and functional success (defined as improvement in visual acuity to better than 6/60 on Snellen’s visual acuity chart from baseline visual acuity).

**Results:**

In conventional group, anatomical success was achieved in 2 (28.6%) eyes, while significant visual improvement was seen in 3 (42.8%) eyes. In Nd:YAG laser embolysis group, anatomical success was achieved in 5 (71.4%) eyes, while significant visual improvement was seen in 6 (85.7%) eyes. All eyes which showed functional improvement underwent Nd:YAG laser embolysis within 6 hours of onset of symptoms.

**Conclusions:**

Nd: YAG laser embolysis is more effective in management of fovea threatening retinal artery occlusion, as compared to conventional medical treatment, if performed within six hours of onset of symptoms.

## INTRODUCTION

Ophthalmic artery is responsible for perfusion of eye. Central retinal artery is an end artery, supplying nerve fibers of retina, as well as inner layers of retina. Occlusion of central retinal artery was first described by von Graefes in 1859, and is responsible for sudden visual loss in affected eye.^1^ There are many clinical conditions associated with development of central retinal artery occlusion like diabetes, hypertension, hyperlipidemia, stroke, smoking, arrhythmias and tumours.^2^ Time is of essence, as prompt diagnosis and appropriate management can save useful vision in eyes suffering from retinal artery occlusion.^3^

Multiple studies have estimated that first few hours are essential in management of retinal artery occlusion, and prognosis is guarded in cases presenting late.^4^ Diagnosis is prompted by the sudden onset of visual acuity loss and the presence of retinal whitening. There is always associated and corresponding field defect. The affected blood vessel shows sluggish blood-flow (boxcarring of the blood column). Careful examination may reveal presence of emboli in major retinal arteries.^5^ Multiple etiologies have been put forward including phacoemulsification surgery, spine surgery, giant cell arteritis, cardiac diseases and other chronic systemic disorders.^6^ Initial management of retinal artery occlusion includes intraocular pressure lowering, ocular massage, promotion of vasodilatation, and anterior chamber paracentesis.^7^ Neodymiu-Doped Yttrium Aluminum Garnet (Nd:YAG) laser embolysis is reported to be effective treatment in management of retinal artery occlusion, where emboli can be visualized easily.^8^ There is limited research available in international and national literature regarding efficacy of conventional medical treatment methods, as well as efficacy of Nd:YAG laser embolysis. The reported efficacy of the procedure is debatable, but it is definitely superior to observation alone in management of this sight threatening disorder.^9^ Rationale of study was to ascertain efficacy of laser intervention as a tangible parameter. The aim of this study was to evaluate the anatomical and functional success after Nd:YAG laser embolysis in retinal artery occlusion patients.

## METHODS

This randomized control trial was carried out at AFIO Rawalpindi from July 2018 to May 2020, after approval (Ref# 194/ERC/AFIO Dated July 9, 2018) from the institutional ethical review committee, and taking written informed consents from patients. Non-probability convenient sampling technique was sued. A total of 14 eyes of 14 patients were analyzed. All patients meeting the required inclusion criteria were randomly divided in two groups using lottery method. Group-A received conventional medical treatment for management of retinal artery occlusion. Group-B was Nd:YAG embolysis group, which received laser treatment for embolysis. Patients from either gender, aged 20-60 years, with fovea threatening or involving retinal artery occlusion (hemiretinal artery or major branch retinal artery) presenting within 24 hours of onset of symptoms were included. Patients with glaucoma, diabetic retinopathy, history of tuberculosis, presentation after 24 hours of onset of symptoms, tilted disc, family history of glaucoma, hereditary optic neuropathies, ocular trauma, ocular surgery, chronic topical steroids users, and previous laser photocoagulation were excluded. Demographic data of study population was acquired. All patients underwent detailed systemic and ophthalmic examination with measurement of best corrected visual acuity, anterior and posterior segment examination, measurement of intraocular pressure, visual field assessment if possible and fundus examination with non-contact fundus viewing lens. Immediately after establishing diagnosis of retinal artery occlusion and visible embolus, initial management protocol was started in all patients including ocular massage, initiation of anti-glaucoma, sublingual glyceryl trinitrate and anterior chamber paracentesis to lower the intraocular pressure. Presence of embolus was confirmed using Optical Coherence Tomography Angiography (OCT-Angio) in all cases. In Group-B, after confirming the presence of embolus on FFA, an Nd:YAG laser embolysis was performed using Goldmann three mirror, Volk Quadraspheric lens, or Volk Trans Equator lens, starting at 0.5mJ and increasing power by 0.5mJ till 2mJ. Maximum power of 2mJ was used. Post procedure, all patients received detailed systemic evaluation by cardiologist and medical specialist to ascertain etiology of event. Visual acuity was checked at one week, one month and three months after procedure. OCT-Angio was also performed at four weeks after laser, if vitreous hemorrhage had resolved. All patients remained on long term follow-up for evaluation of fundus for ischemic changes. All examination and laser was done by single vitreo-retinal surgeon to exclude bias. Anatomical success was defined as restoration of normal flow in retinal artery after 4 weeks of treatment, confirmed by OCT-Angio. Functional success was defined as improvement in best corrected visual acuity to 6/60 or better on Snellen’s visual acuity chart. The pre devised proforma was completed by researcher endorsing subject’s demography and ocular examination findings. Confidentiality of the patient’s record was maintained. Statistical Package for Social Sciences (SPSS 20.0) for windows was used for statistical analysis. Descriptive statistics i.e. mean ± standard deviation for quantitative values (age, presenting hours) and frequencies along with percentages for qualitative variables (gender, laterality of eyes, anatomic success, and functional success) were used to describe the data.

## RESULTS

**Fig.1:**
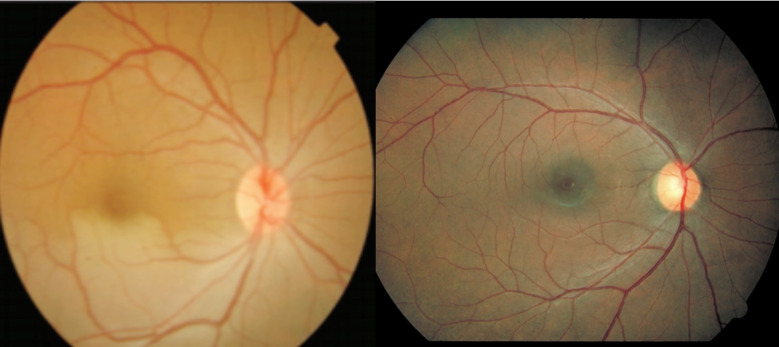
Fundus photograph of a patient with Hemiretinal artery occlusion, before and after YAG Embolysis.

**Fig.2:**
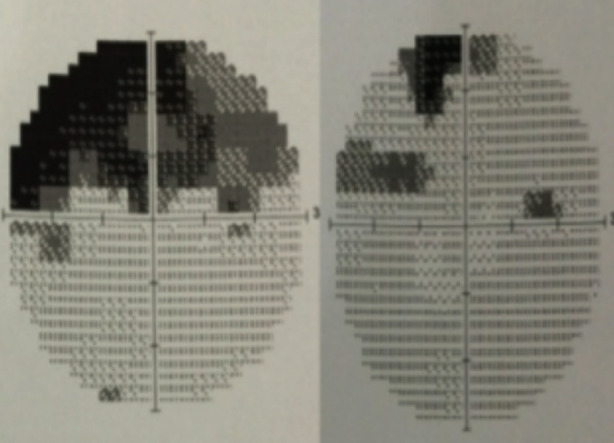
Visual Field testing of same patient before and after laser treatment.

A total of 14 eyes of 14 patients were analyzed. Mean age of study population was 44.63 ± 4.27 years (Range: 40-50 years). Out of 14 patients, 8 (57.14%) subjects were males, while six (42.86%) were females. Mean axial length of study population was 23.71 ± 0.59 mm (Range: 22.2-24.5 mm). Right eye was involved in 7 (50%) patients, while left in 7 (50%) patients. Mean age of patients in Group-A and B was 43.18±3.79 years, and 44.12±4.11 years respectively. Mean axial length in Group-A and B was 23.22 ± 0.65 mm and 23.79±0.85 mm respectively. In conventional group, anatomical success was achieved in 2 (28.6%) eyes, while significant visual improvement was seen in 3 (42.8%) eyes. In Nd:YAG laser embolysis group, anatomical success was achieved in 5 (71.4%) eyes, while significant visual improvement was seen in 6 (85.7%) eyes. Visual acuity profile and anatomical/functional success of all cases in both groups is given in Table-I. All eyes which showed functional improvement underwent Nd:YAG laser embolysis within six hours of onset of symptoms. Mean time of presentation to hospital after initiation of symptoms was 17.23±5.27 hours. All eyes which showed functional improvement underwent Nd:YAG laser embolysis within 6 hours of onset of symptoms. Fundus photograph of one eye showing hemiretinal branch retinal artery occlusion is shown in Fig.1. Visual field testing of same eye, before and after laser treatment is shown in Fig.2.

**Table-I:** Anatomical / Functional success in Study population (n=14).

	Pre-Treatment BCVA(Snellen’s)	Post-Treatment BCVA(Snellen’s)	Functional Success Achieved(Yes/No)	Anatomical Success Achieved(Yes/No)
Group-A (Conventional treatment group)				
Case 1	HM	6/60	Yes	No
Case 2	PL Positive	PL Positive	No	No
Case 3	HM	6/36	Yes	Yes
Case 4	HM	PL Positive	No	No
Case 5	2/60	6/18	Yes	Yes
Case 6	HM	HM	No	No
Case 7	PL Positive	PL Positive	No	No
**Group 2 (Nd:YAG Laser Embolysis Group)**				
Case 1	HM	6/12	Yes	Yes
Case 2	PL Positive	HM	No	No
Case 3	HM	6/60	Yes	Yes
Case 4	HM	6/36	Yes	Yes
Case 5	3/60	6/9	Yes	Yes
Case 6	HM	6/60	Yes	No
Case 7	2/60	6/36	Yes	Yes
HM=Hand movement, PL=Perception of light.				

## DISCUSSION

We evaluated the functional success by improvement in visual acuity to minimum of 6/60 on Snellen’s visual acuity chart. Out of five eyes not showing visual improvement, one improved to 4/60 visual acuity, while one to 2/60 visual acuity on Snellen’s chart. Three eyes did not show any improvement in final visual acuity after three months. In a study conducted on two cases in year 2002, visual acuity significantly improved in both cases.^10^ However, another study found out that visual improvement was not more than 6/60 in cases treated with laser embolysis.^11^ It can be appreciated that in former study, cases with branch retinal artery occlusion were managed, and in later study, poor result can be attributed to central retinal artery involvement. Our study had included only branch retinal artery occlusion and hemiretinal artery occlusion cases to be able to perform laser embolysis, as achieving laser embolysis is not possible in central retinal artery occlusion cases.

We found out that anatomical success was seen in two eyes out of seven in Group-A, while five out of seven in Group-B. In embolysis group, the embolus was not evicted in one case. This case had multiple fibrin platelet type emboli, secondary to carcinoma breast. One of the patient in which anatomical success was not achieved had severe vitreous hemorrhage after laser embolysis refractory to resolution in three months, precluding further retinal view. The similar results of retinal reperfusion were found by researchers in a case series.^12^ In another study, it was found that treated artery trunk and its branches exhibited completely restored blood flow in 38.2% of the cases, basic recovery in 32.4% of the cases, and partial recovery in 14.7% of the cases; the treatment was ineffective in five eyes (14.7%).^13^ This however combined use of intravenous urokinase alongside Nd:YAG laser embolysis for management of retinal artery occlusion. Our results are in line with available data.

The time factor for management of retinal artery occlusion disease is of paramount importance. We found that all cases showing significant visual improvement reported within six hours of onset of symptoms. Patients presenting with central retinal artery occlusion are usually offered treatment even if the time from onset of occlusion is believed to be many hours when there is uncertainty regarding completeness and duration of the occlusion. Patients with incomplete occlusions are more likely to benefit from therapy.^14^ It has been observed by researchers that if complete central retinal artery occlusion is confirmed by observation of segmentation and stagnation of retinal arterial blood flow for longer than 15 minutes on successive fundus examinations, treatment is likely to be futile because of inner retinal infarction.^15^ However, it is not possible to be certain of the prior duration of a complete central retinal artery occlusion observed on a single fundus examination and a variable degree of reperfusion may occur at any time and intermittently.^16^ Even a cherry red spot is not a reliable sign of complete occlusion with retinal infarction because it may occur from inner retinal ischemia without infarction.^17^ It is thus imperative that all patients received in ophthalmic emergency be meticulously examined and treatment initiated to save vision.

We observed that in cases where reperfusion was achieved, vitreous hemorrhage resulted. This was achieved in 5 out of 7 cases in Group-B. This lead to initial drop in visual acuity, which stabilized at three months. The vitreous hemorrhage is mild, intragel hemorrhage, which resolves quickly in absence of any other vascular retinal pathology. In another study, it was reported that vitreous hemorrhage along with aneurysm formation was seen as a result of Nd:YAG laser embolysis.^18^ In the largest data analysis, it was observed that In a weighted analysis vitreous/sub-retinal hemorrhage was estimated to occur in 54% of cases and required vitrectomy in 18% of cases.^19^ However, another case report revealed no complication of said procedure.^20^ It is thus assumed that higher power, and involvement of central retinal artery or large embolus may predispose increased vitreous hemorrhage.

We observed that retinal reperfusion was not achieved in two patient of embolysis group, and five patients in medical group. This was observed in a case with metastasis from breast, and other cases where either higher energy did not result in embolus eviction or vitreous hemorrhage precluded the retinal view. It has been reported that anatomical success is best achieved if less energy used, and case of branch retinal artery occlusion is there, as compared to central retinal artery. Also, the correction in visual acuity is attributed to presenting visual acuity.^21^

Further studies and analysis in this respect will ascertain the efficacy of this procedure in management of central or branch retinal artery occlusion.

### Limitations of the study

Our study has limitations of small sample size, inclusion of branch retinal and central retinal artery occlusion cases only and relatively short follow up of three months. Long term complications related to procedure were also not evaluated.

## CONCLUSIONS

We conclude that Nd:YAG laser embolysis is safe and effective method to regain useful vision in cases of retinal artery occlusion. The literature shows that it is safe procedure, with manageable adverse effects and is definitely superior to observation in cases of retinal artery occlusion. Large cohorts and longer follow ups can provide more meaningful data for application of this procedure as primary therapeutic modality in cases with visible emboli occluding branch or central retinal artery.

### Authors’ Contribution:

**AK** did statistical analysis and manuscript editing.

**AM** conception and design of study, manuscript writing, approval of manuscript.

**MAM** did data acquisition, data analysis, manuscript writing.
